# An Uncommon Presentation of Hypertensive Emergency in a Young Patient: A Case Report

**DOI:** 10.7759/cureus.113317

**Published:** 2026-07-24

**Authors:** Jacqueline Tran, Matan S Malka, Mark Curato

**Affiliations:** 1 Emergency Medicine, Weill Cornell Medicine, New York, USA

**Keywords:** acute kidney injury, hypertensive emergency, hypertensive retinopathy, thrombotic microangiopathy, young adult hypertension

## Abstract

A hypertensive emergency is defined as severe blood pressure elevation with acute end-organ damage, carrying significant morbidity and mortality.

We describe a 25-year-old male with no prior medical history of hypertension who presented with painless hematuria, epistaxis, mild headache, and unilateral upper extremity paresthesia. Initial blood pressure was 253/167 mmHg. The patient’s workup was notable for evidence of acute kidney injury, hematuria, and rising troponins. Electrocardiogram demonstrated ST elevations in aVR and V1-V3, with diffuse ST depressions. Computed tomography (CT) scans were negative for aortic dissection and intracranial pathology. Given evidence of end-organ damage, he was diagnosed with hypertensive emergency and treated with intravenous labetalol followed by nicardipine infusion, targeting a 20-25% reduction in blood pressure. Further evaluation revealed hypertensive retinopathy and renal thrombotic microangiopathy on biopsy. Workup for secondary causes was pursued, and he was transitioned to multidrug oral antihypertensive therapy with subsequent blood pressure control and outpatient follow-up.

In conclusion, this case highlights an uncommon presentation of hypertensive emergency in a young patient without prior history of hypertension. It emphasizes the importance of recognizing end-organ damage rather than focusing solely on numeric blood pressure values.

## Introduction

Hypertension is a chronic illness that affects approximately 46% of adults in the United States. The chief complaint of elevated blood pressure accounts for 0.3-0.6% of all ED visits [1], and 25-36% of those patients will have a hypertensive emergency, which is defined by the American Heart Association as a systolic blood pressure (SBP) over diastolic blood pressure (DBP) of greater than 180/110-120mmHg, with evidence of end-organ damage [2]. This is distinct from hypertensive urgency, in which individuals may have severely elevated blood pressure without signs of end-organ damage. Regardless of the terminology, the emphasis should be placed on mitigating the potential sequelae of acute or severe hypertension rather than the number itself. Signs and symptoms of end-organ damage in hypertensive emergency include acute pulmonary edema, stroke, cardiac ischemia, congestive heart failure, encephalopathy, and acute kidney injury. In a large systematic review from 2023, ischemic stroke and pulmonary edema were the most common sequelae, representing 24% and 28% of patients with hypertensive emergency, respectively [3].

A hypertensive emergency carries a high hospitalization and mortality rate, making early identification and management critical. In a 2021 cross-sectional study at a tertiary care center, 40% of patients deemed to have a hypertensive emergency were admitted and found to have 1-year mortality rates of 26.9% [4]. In a 2023 systematic review of clinical outcomes of hypertensive emergency, 84% of all patients diagnosed with hypertensive emergency were admitted, and 10% died during their hospitalization [3]. Those presenting with hypertensive emergency are typically adults >60 years old, male, and carry diagnoses of hypertension or comorbidities such as diabetes, chronic kidney disease, or cardiovascular disease. Medication non-adherence, sympathomimetic drug use, and dietary indiscretion are common triggers for hypertensive emergency in these patients.

Only approximately 23% of hypertensive emergencies occur in patients with no known diagnosis of hypertension [5]. We present a rare case in which a young patient without a prior diagnosis or any known risk factors presented to our emergency department and was found to be in a hypertensive emergency.

## Case presentation

Emergency department course

**Figure 1:**
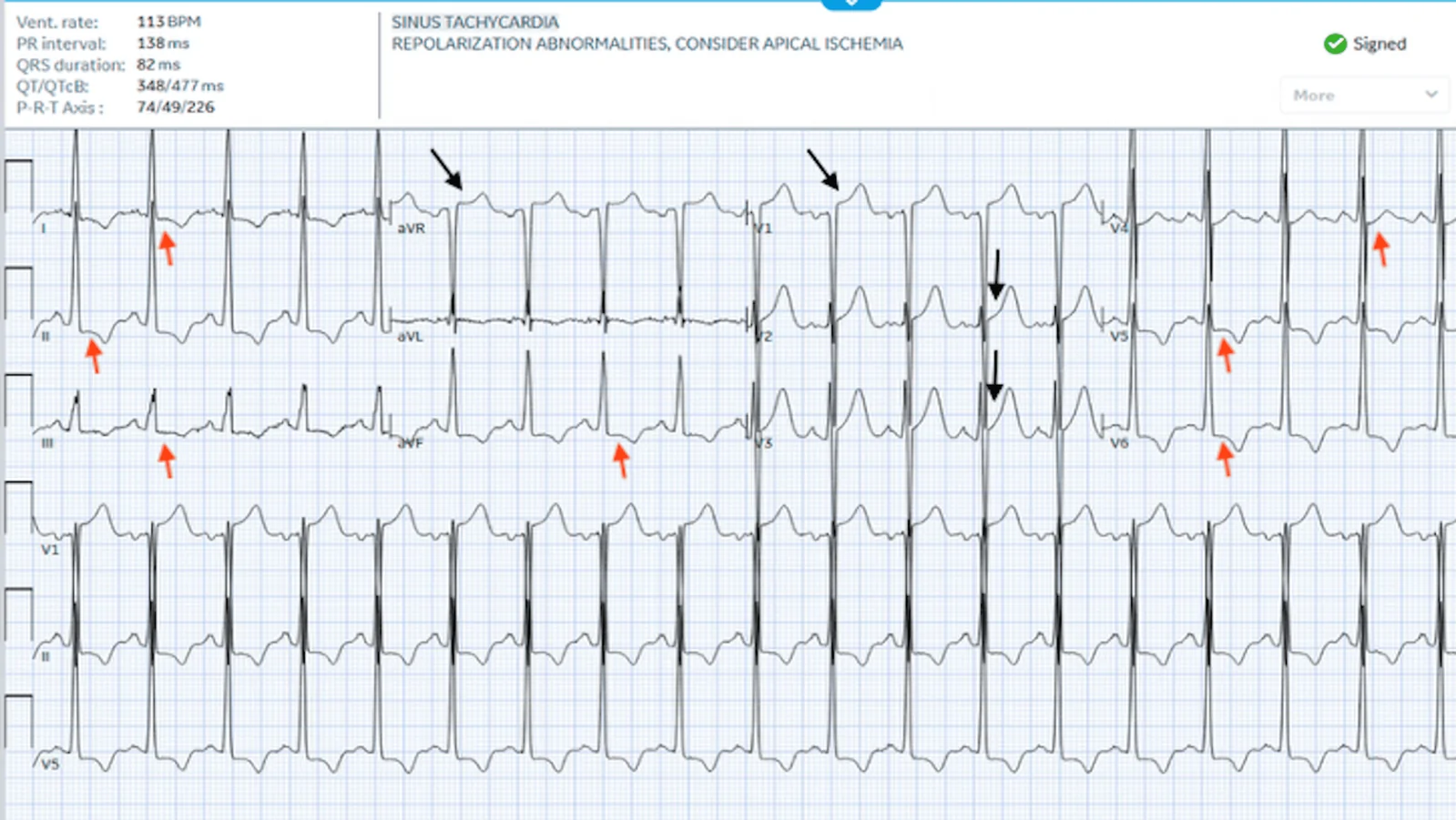
Initial EKG on arrival Black arrows indicate ST elevations; Red arrows indicate ST depressions

A 25-year-old male with the sole medical history of psoriasis presented to the Emergency Department with a chief complaint of painless hematuria for one week, a single episode of epistaxis, and mild headache since that morning. The patient further reported left upper extremity paresthesia for the past week. On further history, he reported he has not seen a doctor since his last pediatrician visit six years earlier. He had no prior history of smoking or recreational drug or alcohol use. His vital signs upon arrival were notable for a blood pressure of 253/167 and a heart rate of 114. His SpO2 was 99%, and body temperature was 37.2 °C. An EKG was performed, notable for ST elevations in aVR and V1-V3, with diffuse ST depressions (Figure 1). 

**Figure 2:**
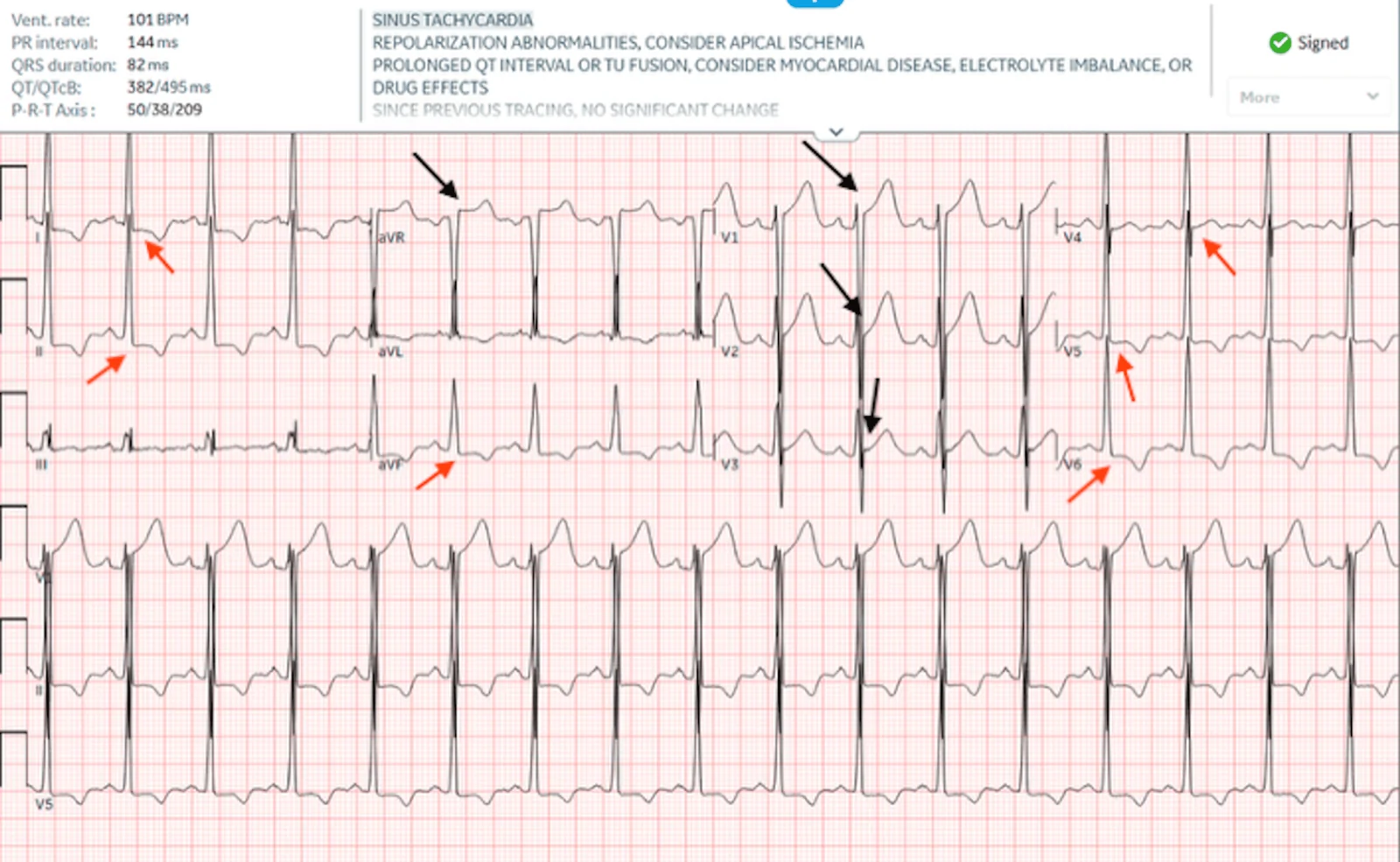
Second EKG 1.5 hours after the initial Black arrows indicate ST elevations; Red arrows indicate ST depressions

On physical exam, the patient was in no acute distress. Subjective decreased sensation in the left upper extremity was noted. However, there was 5/5 strength, which was symmetric in all four extremities; ambulation was with a steady gait, there was no dysmetria, and no pronator drift. Laboratory evaluation was notable for a creatinine of 2.18 with a blood urea nitrogen (BUN) of 28, urinalysis with RBC >182, and high-sensitivity troponins of 34, 35 (1 hour), and 38 (2 hours). CBC and LFTs were within normal limits. A second EKG done an hour and a half later showed no major changes (Figure 2). The patient underwent non-contrast CT of the head revealing no ​acute intracranial hemorrhage, territorial loss of gray-white matter differentiation, or mass effect. Given normal CT of the head and the reassuring neurological exam, there was lower suspicion for large-vessel occlusion. Thrombotic thrombocytopenic purpura was also excluded, as the patient had a normal hemoglobin, normal platelet count, normal bilirubin, and no skin findings. Similarly, hemolytic uremic syndrome was also excluded given no lab findings indicating hemolysis. A CT angiogram of the chest, abdomen, and pelvis was performed, with no acute aortic process or acute finding. He was diagnosed with hypertensive emergency. He was treated with labetalol 10 mg IV and a nicardipine infusion with a target goal of reducing his blood pressure by 20%. To trend the blood pressure, he was placed on a continuous monitor, measuring his blood pressure every 15 minutes after being started on the nicardipine infusion. Table 1 exhibits the charted blood pressure measurements. While in the emergency department, his blood pressure remained in the 190s/130s, and he was admitted to the cardiac intensive care unit (CCU).

**Table 1:** Charted blood pressure measurements in the emergency department

Length of Stay (hours: minutes)	Blood Pressure (mmHg)
00:00	253/167
00:59	231/156
2:02	220/149
2:38	191/133
2:57	202/135
3:06	192/128
3:37	215/147
4:22	202/145
4:37	198/136

CCU course

The patient’s troponin peaked at 75 on hospital day 2. Workup for secondary causes of hypertension was pursued; cortisol levels were not suggestive of Cushing’s, but RNA polymerase III (an auto-antibody specific for scleroderma with renal involvement) was positive, and his elevated creatinine and urine protein were suggestive of renal parenchymal disease. Further workup for glomerulonephropathy, including erythrocyte sedimentation rate, complement levels, HIV, hepatitis panel, anti-neutrophil cytoplasmic antibodies, double-stranded DNA, and anti-glomerular basement membrane, was negative. The patient underwent transthoracic echocardiography, which revealed concentric left ventricular hypertrophy, normal function in both ventricles, and a normal ejection fraction of 60%. Bilateral renal ultrasound did not reveal renal artery stenosis. A CT of the abdomen and pelvis did not reveal adrenal masses. He was diagnosed with grade 3 hypertensive retinopathy on fundoscopic exam. The patient underwent a renal biopsy on hospital day 5, which was complicated by a hematoma. The result was for renal thrombotic microangiopathy (TMA), which can be autoimmune-mediated, coagulation-mediated, drug- or toxin-induced, or metabolism-associated. On day 3, he began oral treatment with amlodipine, hydrochlorothiazide, and losartan. He was transitioned off the nicardipine infusion on hospital day 8 and transferred to the general medical floor on day 8.

Floor course

THE oral antihypertensives were continued, and he was discharged on hospital day 9 on amlodipine 10 mg daily, hydrochlorothiazide 50 mg daily, losartan 100 mg daily, carvedilol 25 mg BID, and eplerenone 50 mg BID. He was instructed to follow up on an outpatient basis with nephrology, cardiology, and ophthalmology.

Post-hospitalization 

After discharge, the patient was compliant with follow-up and asymptomatic. Blood pressure was controlled at 110-120 systolic. Consideration was given to initiating ravulizumab or eculizumab for treatment of severe TMA, and he was to undergo genetic testing. 

## Discussion

This case represents an uncommon presentation of hypertensive emergency with several atypical features relevant to emergency physicians (Table 2). Only 8-23% of hypertensive emergencies occur in patients without previously known hypertension [5,6]. Furthermore, a hypertensive emergency is more common in persons older than 60 years of age [7], and rare in patients such as this 25-year-old. The patient's constellation of symptoms--painless hematuria, epistaxis, mild headache, and left arm numbness--demonstrates the diverse manifestations of end-organ damage that can occur in hypertensive emergency, including acute kidney injury, hypertensive retinopathy, and neurologic symptoms [2,3]. This case underscores the importance of maintaining a high index of suspicion for hypertensive emergencies in young adults presenting with multisystem symptoms, even without a prior diagnosis of hypertension, and emphasizes the need for aggressive evaluation of secondary causes in this age group [8,9].

**Table 2:** Comparison of our case’s atypical features with existing literature

Feature:	Typical Hypertensive Emergency	Present Case
Age	>60 years of age [7]	25 years old
Prior Diagnosis of Hypertension	77-92% of patients have known history [5,6]	No prior history of hypertension
Risk Factors	Illicit drug use, current smokers, history of chronic kidney disease [2,7]	No risk factors
Presenting Symptoms	Headache, chest pain, dyspnea, dizziness, epistaxis [2]	Painless hematuria, epistaxis, mild headache, and left arm numbness
End-Organ Damage Pattern	Pulmonary edema (28%), ischemic stroke (24%) [3]	Renal failure, cardiac ischemia, retinopathy

Emergency department recognition and initial management 

The patient's presentation exemplifies the importance of recognizing end-organ damage rather than focusing solely on numeric blood pressure [2,7,10,11]. With blood pressure of 253/167 mmHg and evidence of acute kidney injury (creatinine 2.18), cardiac ischemia (troponin elevation, EKG changes), and hematuria, this patient met criteria for hypertensive emergency requiring immediate intervention. The 2025 AHA/ACC guidelines recommend reducing blood pressure by 25% in the first hour, followed by gradual further reduction over the next 24-28 hours [2]. Overly aggressive blood pressure reduction can result in vital organ hypoperfusion due to loss of autoregulation [2,7].

In this case, the initial treatment was with labetalol IV, followed by nicardipine infusion. Nicardipine is a preferred agent for a hypertensive emergency with acute kidney injury, offering titratable blood pressure control with rapid onset and no negative inotropic effects [2,7]. Studies have shown faster achievement of blood pressure control and less variability with nicardipine compared to labetalol [2,7]. The target of 20% reduction was appropriate for this patient's presentation.

Secondary hypertension evaluation in young adults 

The patient's young age without known hypertension mandated aggressive evaluation for secondary causes. Approximately 29.6% of hypertensive patients aged 18-40 years have identifiable secondary causes, with primary aldosteronism being most common (54.8%), followed by renovascular hypertension (18.4%) and primary kidney disease (12.9%) [9]. Importantly, the prevalence of secondary hypertension in young adults is high, regardless of blood pressure level, emphasizing that all young patients with hypertension warrant screening [8,9,12].

Disposition and prognosis

Hypertensive emergencies carry significant morbidity and mortality [2,3]. All patients with a hypertensive emergency should be admitted to an intensive care unit for continuous blood pressure monitoring and intravenous antihypertensive therapy [2,7]. Chronic kidney disease is present in 79% of patients admitted with acute severe hypertension, and any degree of acute kidney injury is associated with a greater risk of morbidity and mortality, with significantly higher 90-day mortality [13-16].

## Conclusions

This case illustrates that a hypertensive emergency can present in young adults without a prior history of hypertension, reinforcing the need for emergency physicians to maintain a high index of suspicion when multisystem symptoms accompany severely elevated blood pressure. Clinical decision-making should prioritize the identification of end-organ damage over reliance on numeric blood pressure. Early recognition, appropriate blood pressure management with a target reduction of 25% in the first hour, and prompt evaluation for secondary causes of hypertension are essential to improving outcomes in this patient population.
